# Supplementary material for: Rheum rhaponticum and Rheum rhabarbarum Extracts as Modulators of Endothelial Cell Inflammatory Response
Source: Nutrients. 2023 Feb 14;15(4):949. doi: 10.3390/nu15040949 (PMC9964395; doi:10.3390/nu15040949)
Supplement: Supplementary file 1 [file nutrients-15-00949-s001.zip › Supplementary materials S1.pdf]

SUPPLEMENTARY MATERIALS S1

Rheum rhaponticum and *Rheum rhabarbarum extracts* As Modulators of Endothelial Cell Inflammatory Response

Oleksandra Liudvytska [1,*], Michał B. Ponczek [1], Oskar Ciesielski [2,3], Justyna Krzyżanowska-Kowalczyk [4], Mariusz Kowalczyk [4], Aneta Balcerczyk [2] and Joanna Kolodziejczyk-Czepas [1]

1 Department of General Biochemistry, Faculty of Biology and Environmental Protection, University of Lodz, 90-236 Lodz, Poland
2 Department of Sociobiology and Epigenetics, Faculty of Biology and Environmental Protection, University of Lodz, 90-236 Lodz, Poland
3 The Bio-Med-Chem Doctoral School, University of Lodz and Lodz Institutes of the Polish Academy of Sciences, University of Lodz, Banacha 12/16, 90-237, Lodz, Poland
4 Department of Biochemistry and Crop Quality, Institute of Soil Science and Plant Cultivation, State Research Institute, Czartoryskich 8, 24-100 Puławy, Poland
* Correspondence: oleksandra.liudvytska@biol.uni.lodz.pl; Tel.: +48-42-635-44-84

Contents:

Figure S1. UHPLC profile of the butanol extract obtained from the petioles of *Rheum rhabarbarum* (upper panel - CAD detector signal, lower panel - MS chromatogram using negative ESI mode, the numbers correspond to the numbers of compounds tentatively identified in Table S1).

Figure S2. UHPLC profile of the butanol extract obtained from *Rheum rhabarbarum* roots/rhizomes (upper panel - CAD detector signal, lower panel - MS chromatogram using negative ESI mode, the numbers correspond to the numbers of compounds tentatively identified in Table S2).

Figure S3. UHPLC profile of the butanol extract obtained from the petioles of *Rheum rhaponticum* (upper panel - CAD detector signal, lower panel - MS chromatogram using negative ESI mode, the numbers correspond to the numbers of compounds tentatively identified in Table 1S).

Figure S4. UHPLC profile of the butanol extract obtained from *Rheum rhaponticum* roots/rhizomes (upper panel - CAD detector signal, lower panel - MS chromatogram using the negative ESI mode, the numbers correspond to the numbers of compounds tentatively identified in Table S2).

Table S1. Comparative analysis of metabolites identified in butanol extracts obtained from the petioles of *Rheum rhaponticum* and *Rheum rhabarbarum*.

Table S2. Comparative analysis of metabolites identified in butanol extracts obtained from the roots/rhizomes of *Rheum rhaponticum* and *Rheum rhabarbarum*.

Table S3. Qualitative similarities and unique metabolites observed between the extracts from petioles (A) and roots (B) of *R. rhabarbarum* and *R. rhaponticum*.

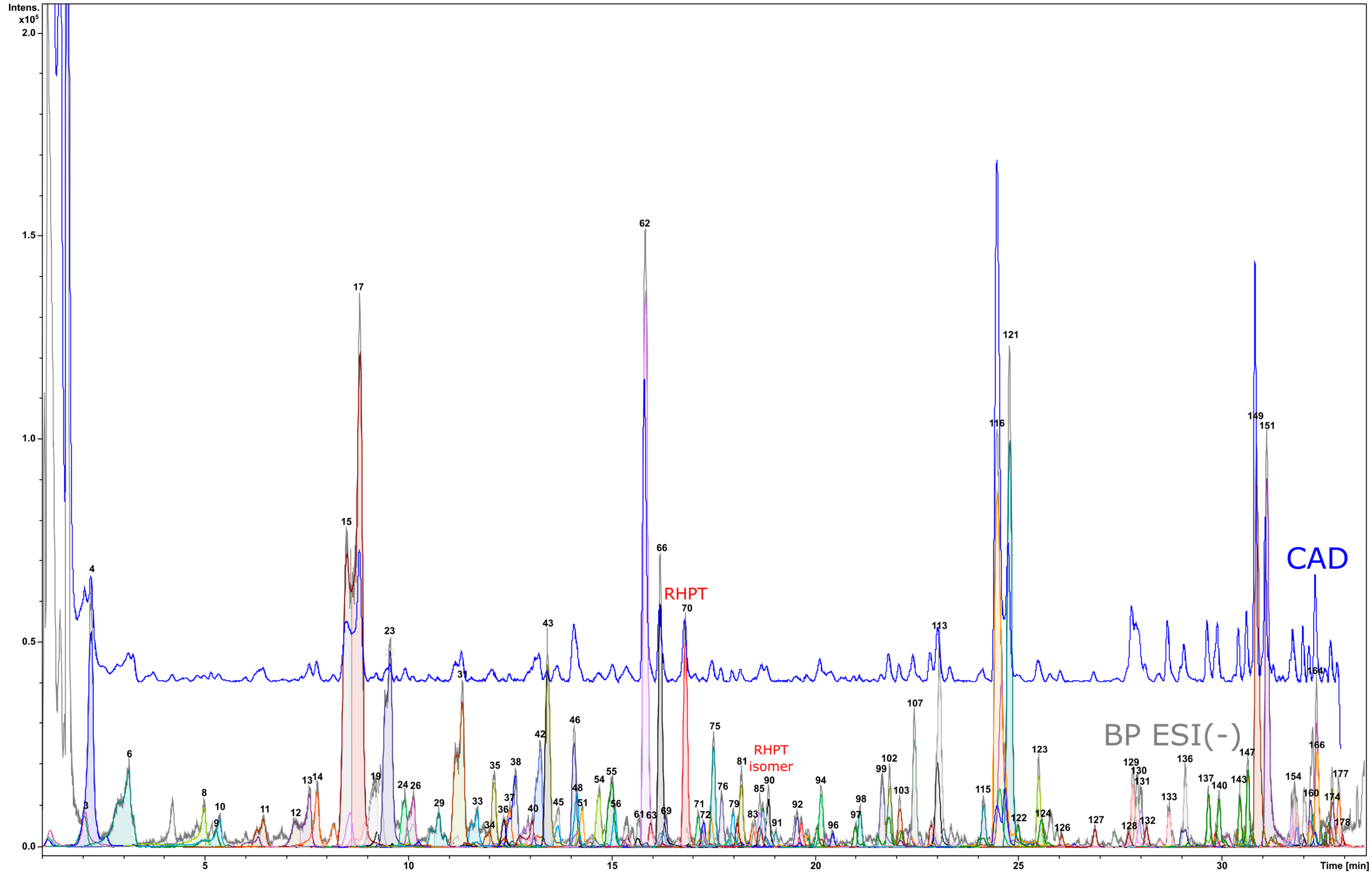

Figure S1. UHPLC profile of the butanol extracts obtained from the petioles of *Rheum rhabarbarum* (upper panel - CAD detector signal, lower panel - MS chromatogram using negative ESI mode, the numbers correspond to the numbers of compounds tentatively identified in Table S1).

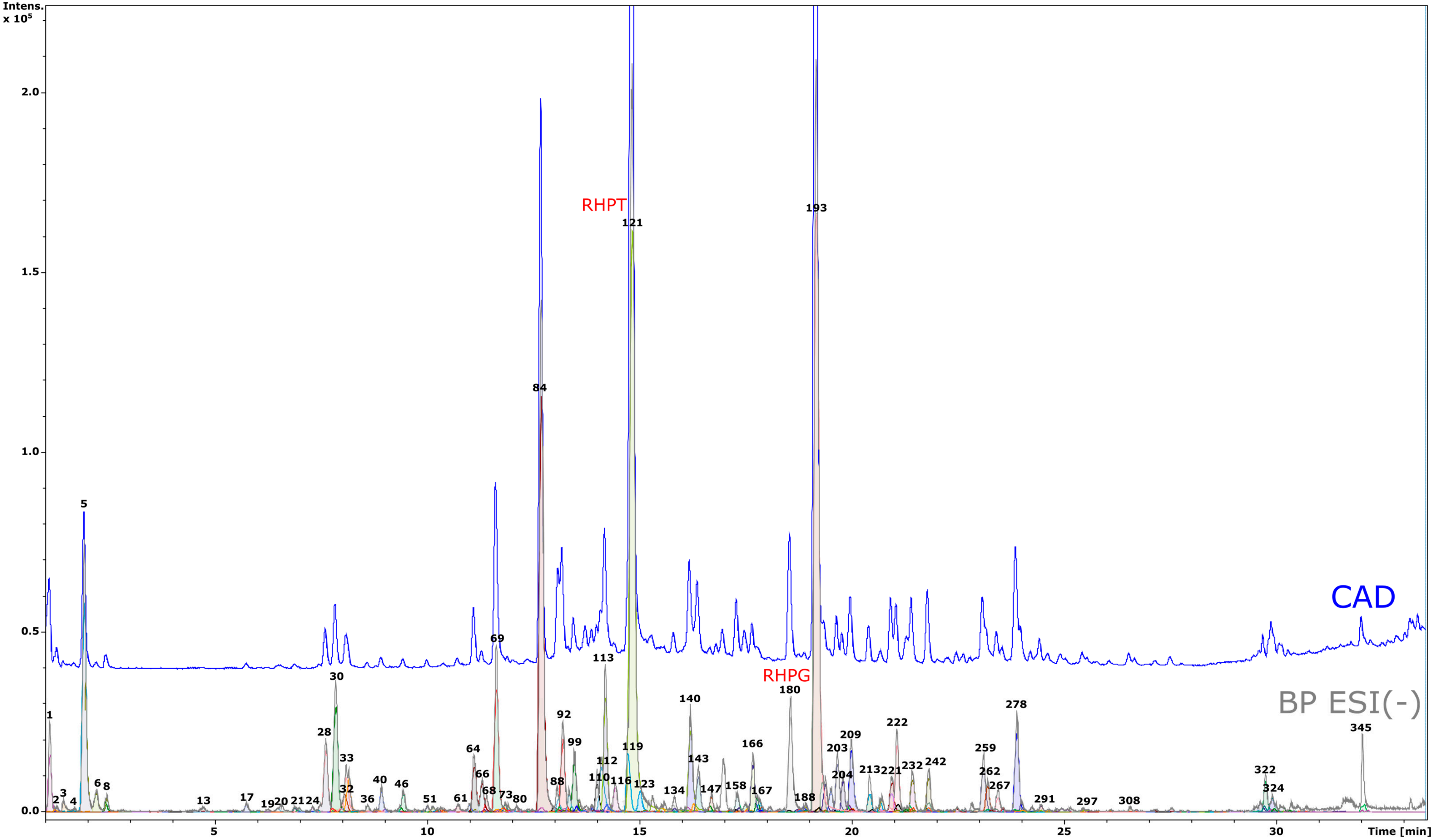

Figure S2. UHPLC profile of the butanol extracts obtained from *Rheum rhabarbarum* roots/rhizomes (upper panel - CAD detector signal, lower panel - MS chromatogram using negative ESI mode, the numbers correspond to the numbers of compounds tentatively identified in Table S2).

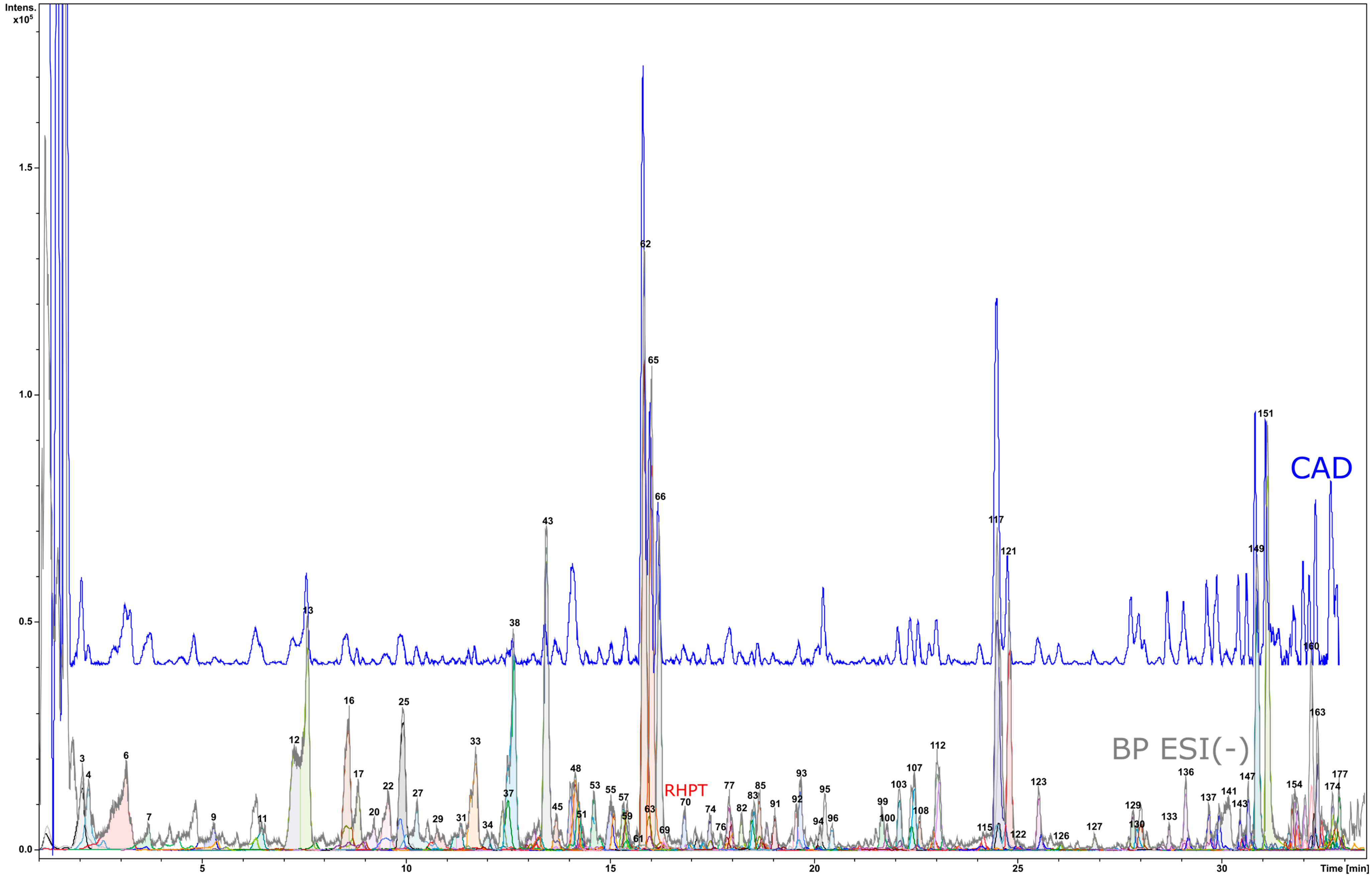

Figure S3. UHPLC profile of the butanol extracts obtained from the petioles of *Rheum rhaponticum* (upper panel - CAD detector signal, lower panel - MS chromatogram using negative ESI mode, the numbers correspond to the numbers of compounds tentatively identified in Table S1).

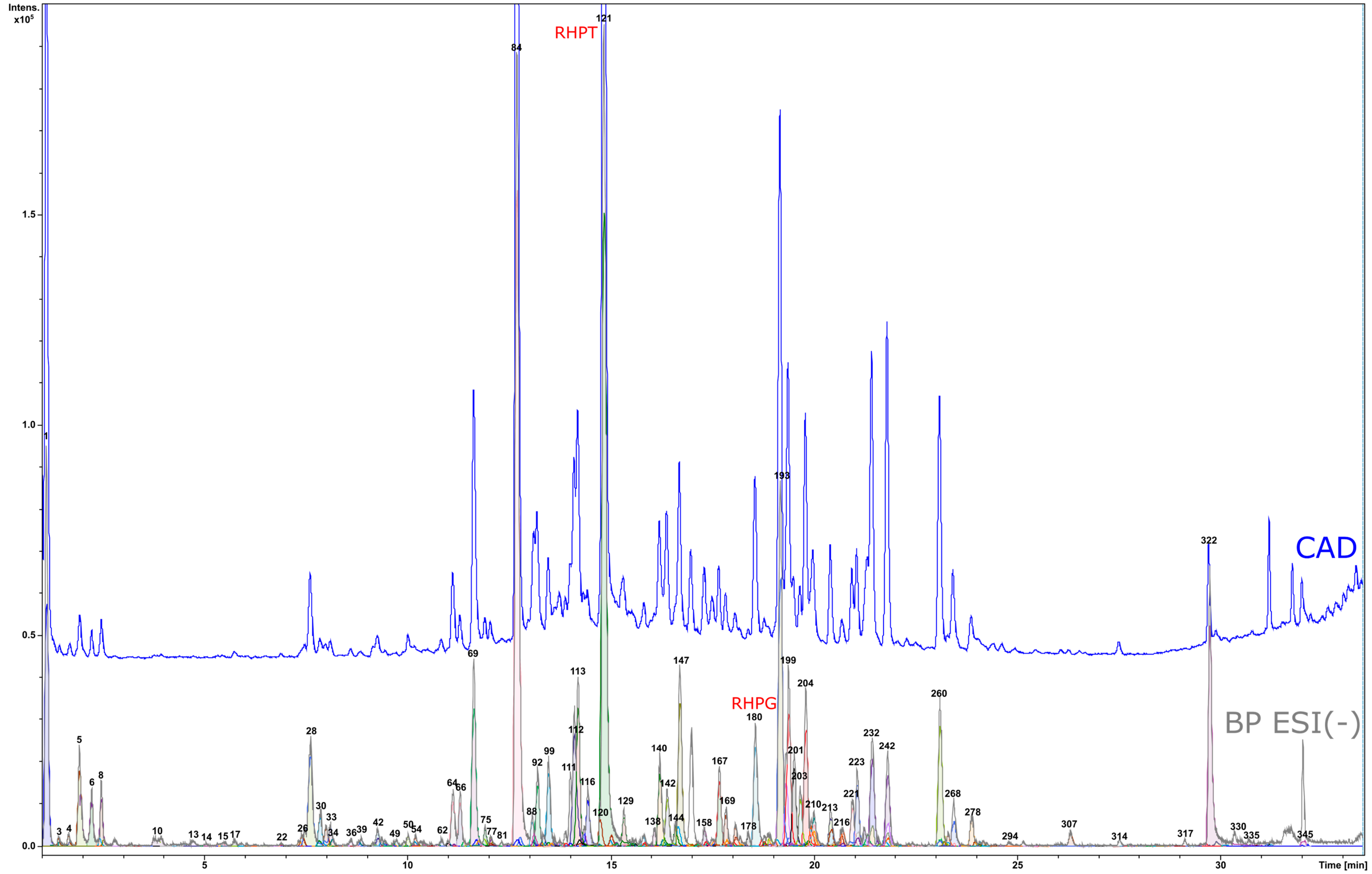

Figure S4. UHPLC profile of the butanol extracts obtained from *Rheum rhaponticum* roots/rhizomes (upper panel - CAD detector signal, lower panel - MS chromatogram using the negative ESI mode, the numbers correspond to the numbers of compounds tentatively identified in Table S2).
