# Supplementary material for: Rheum rhaponticum and Rheum rhabarbarum Extracts as Modulators of Endothelial Cell Inflammatory Response
Source: Nutrients. 2023 Feb 14;15(4):949. doi: 10.3390/nu15040949 (PMC9964395; doi:10.3390/nu15040949)
Supplement: Supplementary file 1 [file nutrients-15-00949-s001.zip › Supplementary materials S2.pdf]

Rheum rhaponticum and Rheum rhabarbarum Extracts As Modulators of Endothelial Cell Inflammatory Response

Oleksandra Liudvytska [1,*], Michał B. Ponczek [1], Oskar Ciesielski [2,3], Justyna Krzyżanowska-Kowalczyk [4], Mariusz Kowalczyk [4], Aneta Balcerczyk [2] and Joanna Kolodziejczyk-Czepas [1]

[1] Department of General Biochemistry, Faculty of Biology and Environmental Protection, University of Lodz, 90-236 Lodz, Poland

[2] Department of Sociobiology and Epigenetics, Faculty of Biology and Environmental Protection, University of Lodz, 90-236 Lodz, Poland

[3] The Bio-Med-Chem Doctoral School, University of Lodz and Lodz Institutes of the Polish Academy of Sciences, University of Lodz, Banacha 12/16, 90-237, Lodz, Poland

[4] Department of Biochemistry and Crop Quality, Institute of Soil Science and Plant Cultivation, State Research Institute, Czartoryskich 8, 24-100 Puławy, Poland

* Correspondence: oleksandra.liudvytska@biol.uni.lodz.pl; Tel.: +48-42-635-44-84

arachidonic acid (substrate for both enzymes)

Swiss Institute of Bioinformatics

Home FAQ Help Download Contact Disclaimer

Query Molecule

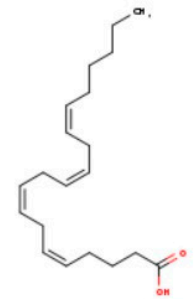

Target Classes

Top 15
Top 25
Top 50
All

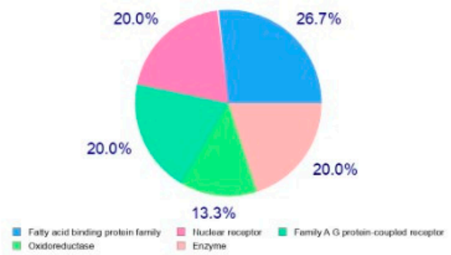

Export results:

Show 15 entries

Search:

Target	Common name	Uniprot ID	ChEMBL ID	Target Class	Probability*	Known actives (3D/2D)
Fatty acid binding protein adipocyte	FABP4	P15090	CHEMBL2083	Fatty acid binding protein family		65 / 3
Peroxisome proliferator-activated receptor gamma	PPARG	P37231	CHEMBL235	Nuclear receptor		641 / 22
Peroxisome proliferator-activated receptor alpha	PPARA	Q07869	CHEMBL239	Nuclear receptor		394 / 13
Peroxisome proliferator-activated receptor delta	PPARD	Q03181	CHEMBL3979	Nuclear receptor		221 / 8
Free fatty acid receptor 1	FFAR1	O14842	CHEMBL4422	Family A G protein-coupled receptor		203 / 2
Fatty acid binding protein muscle	FABP3	P05413	CHEMBL3344	Fatty acid binding protein family		32 / 4
Arachidonate 5-lipoxygenase	ALOX5	P09917	CHEMBL215	Oxidoreductase		121 / 25
Cyclooxygenase-1	PTGS1	P23219	CHEMBL221	Oxidoreductase		17 / 2
Cannabinoid receptor 1	CNR1	P21554	CHEMBL218	Family A G protein-coupled receptor		26 / 102
Anandamide amidohydrolase	FAAH	O00519	CHEMBL2243	Enzyme		10 / 26
Telomerase reverse transcriptase	TERT	O14746	CHEMBL2916	Enzyme		3 / 2
Fatty acid binding protein epidermal	FABP5	Q01469	CHEMBL3674	Fatty acid binding protein family		11 / 1

SIB Swiss Institute of Bioinformatics

SwissTargetPrediction

Home FAQ Help Download Contact Disclaimer

Query Molecule

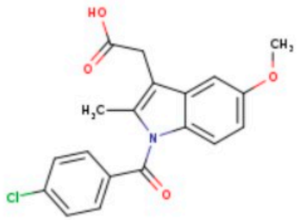

Target Classes

Top 15
Top 25
Top 50
All

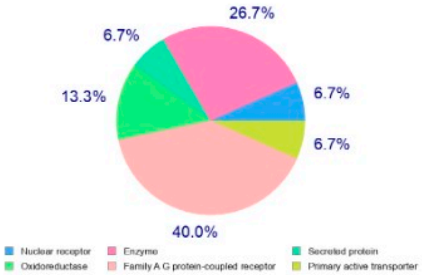

Export results:

Show 15 entries Search:

Target	Common name	Uniprot ID	ChEMBL ID	Target Class	Probability*	Known actives (3D/2D)
Androgen Receptor	AR	P10275	CHEMBL1871	Nuclear receptor		6 / 4
Aldose reductase	AKR1B1	P15121	CHEMBL1900	Enzyme		395 / 12
Interleukin-8	CXCL8	P10145	CHEMBL2157	Secreted protein		12 / 1
Cyclooxygenase-1	PTGS1	P23219	CHEMBL221	Oxidoreductase		59 / 32
Cyclooxygenase-2	PTGS2	P35354	CHEMBL230	Oxidoreductase		155 / 179
Aldo-keto-reductase family 1 member C3	AKR1C3	P42330	CHEMBL4681	Enzyme		172 / 55
G protein-coupled receptor 44	PTGDR2	Q9Y5Y4	CHEMBL5071	Family A G protein-coupled receptor		1115 / 341
Nitric oxide synthase, inducible *(by homology)*	NOS2	P35228	CHEMBL4481	Enzyme		2 / 1
P-glycoprotein 1	ABCB1	P08183	CHEMBL4302	Primary active transporter		1 / 20
Prostanoid DP receptor	PTGDR	Q13258	CHEMBL4427	Family A G protein-coupled receptor		148 / 90

Indomethacin (COX-2 inhibitor)

SwissTargetPrediction

Swiss Institute of Bioinformatics

Home FAQ Help Download Contact Disclaimer

Query Molecule

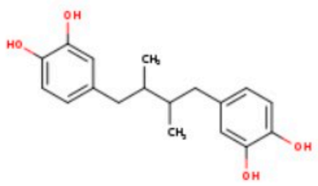

Target Classes

Top 15
Top 25
Top 50
All

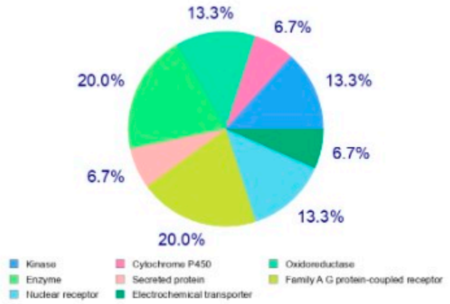

Export results:

Show 15 entries Search:

Target	Common name	Uniprot ID	ChEMBL ID	Target Class	Probability*	Known actives (3D/2D)
Insulin-like growth factor I receptor	IGF1R	P08069	CHEMBL1957	Kinase		23 / 14
Cytochrome P450 19A1	CYP19A1	P11511	CHEMBL1978	Cytochrome P450		43 / 47
Arachidonate 5-lipoxygenase	ALOX5	P09917	CHEMBL215	Oxidoreductase		31 / 18
Arachidonate 15-lipoxygenase	ALOX15	P16050	CHEMBL2903	Enzyme		27 / 17
Transthyretin	TTR	P02766	CHEMBL3194	Secreted protein		2 / 2
Arachidonate 12-lipoxygenase	ALOX12	P18054	CHEMBL3687	Enzyme		10 / 9
Leukotriene B4 receptor 1	LTB4R	Q15722	CHEMBL3911	Family A G protein-coupled receptor		3 / 1
Cyclooxygenase-2	PTGS2	P35354	CHEMBL230	Oxidoreductase		31 / 10
Estrogen receptor alpha	ESR1	P03372	CHEMBL206	Nuclear receptor		360 / 92

NDGA (COX-2 and 5-LOX inhibitor)

SwissDrugDesign | SwissDock SwissParam SwissSidechain SwissBioisostere **SwissTargetPrediction** SwissADME SwissSimilarity | About us

SIB Swiss Institute of Bioinformatics

SwissTargetPrediction

Home FAQ Help Download Contact Disclaimer

Query Molecule

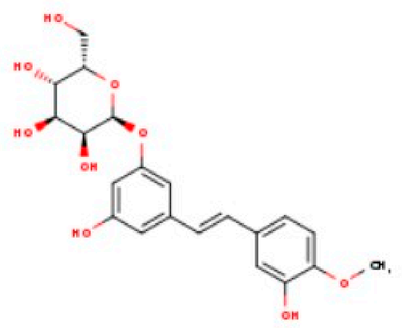

Target Classes

Top 15
Top 25
Top 50
All

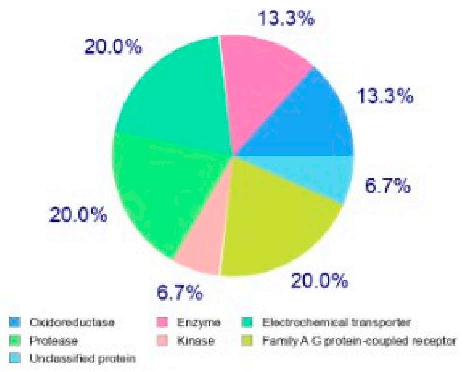

Export results:

Show 15 entries

Search: oxy

Target	Common name	Uniprot ID	ChEMBL ID	Target Class	Probability*	Known actives (3D/2D)
Cyclooxygenase-1	PTGS1	P23219	CHEMBL221	Oxidoreductase		0 / 3
Cyclooxygenase-2	PTGS2	P35354	CHEMBL230	Oxidoreductase		5 / 2
Hydroxycarboxylic acid receptor 2	HCAR2	Q8TDS4	CHEMBL3785	Family A G protein-coupled receptor		45 / 0

rhaponticin
(rhapontin)

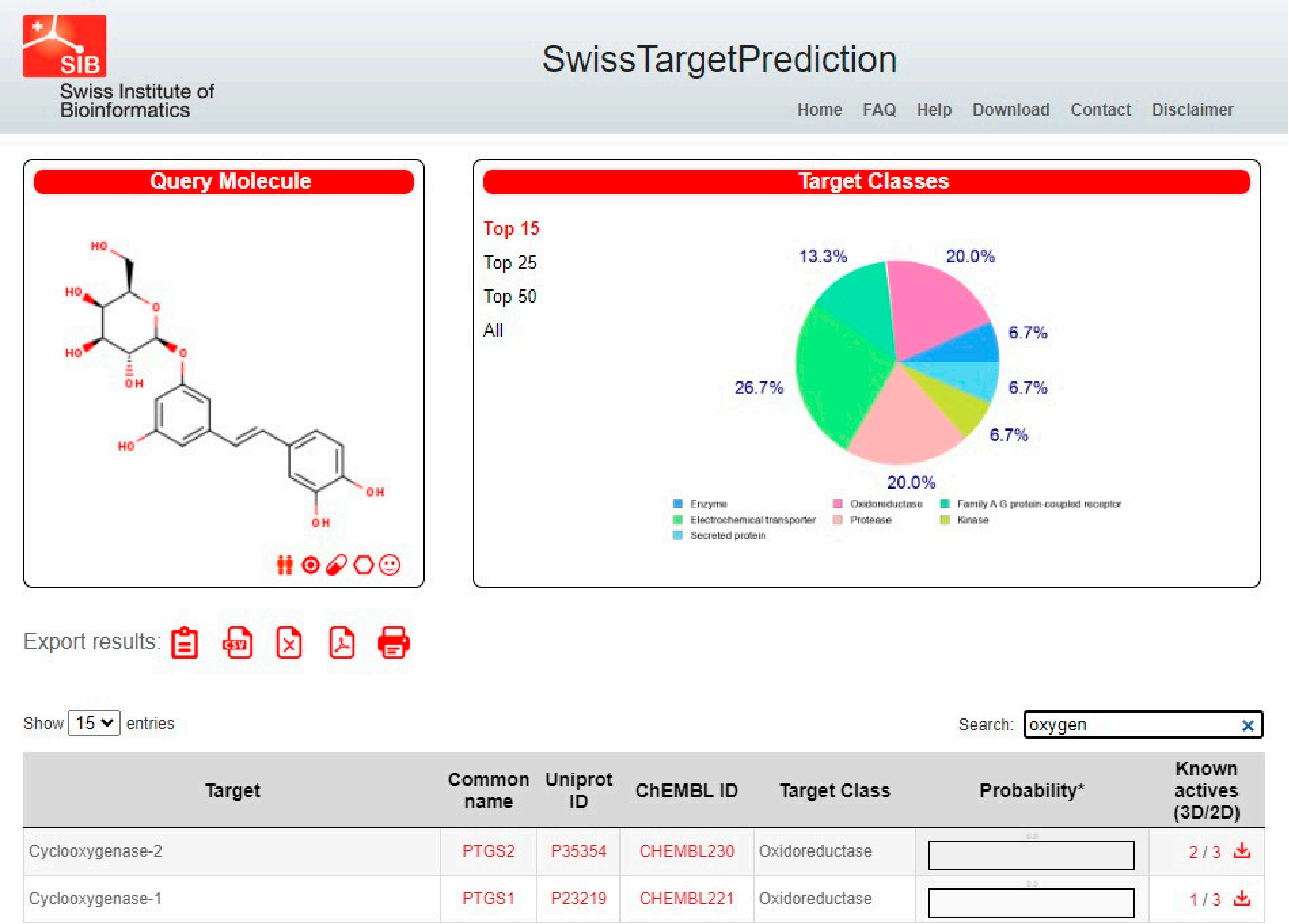
SIB
Swiss Institute of Bioinformatics
SwissTargetPrediction
Home FAQ Help Download Contact Disclaimer
Query Molecule
Target Classes
Top 15
Top 25
Top 50
All
13.3%
20.0%
6.7%
6.7%
6.7%
26.7%
20.0%
Enzyme
Oxidoreductase
Family A G protein-coupled receptor
Electrochemical transporter
Protease
Kinase
Secreted protein
Export results:
Show 15 entries
Search: oxygen
Target | Common name | Uniprot ID | ChEMBL ID | Target Class | Probability* | Known actives (3D/2D)
Cyclooxygenase-2 | PTGS2 | P35354 | CHEMBL230 | Oxidoreductase | | 2 / 3
Cyclooxygenase-1 | PTGS1 | P23219 | CHEMBL221 | Oxidoreductase | | 1 / 3

astringin

SwissTargetPrediction

Swiss Institute of Bioinformatics

Home FAQ Help Download Contact Disclaimer

Query Molecule

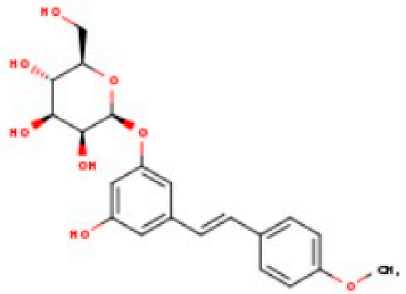

Target Classes

Top 15
Top 25
Top 50
All

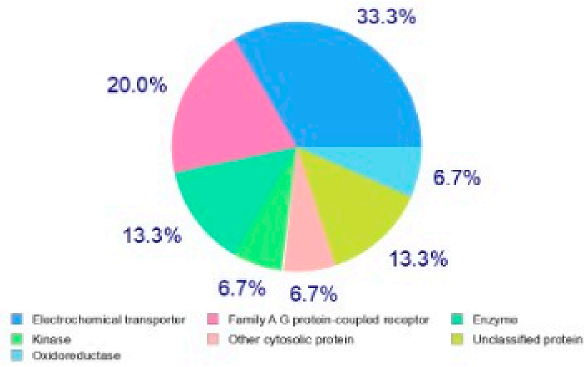

deoxyrhaponticin

Export results:

Show 15 entries

Search: oxy

Target	Common name	Uniprot ID	ChEMBL ID	Target Class	Probability*	Known actives (3D/2D)
Cyclooxygenase-1	PTGS1	P23219	CHEMBL221	Oxidoreductase		0 / 4
Cyclooxygenase-2	PTGS2	P35354	CHEMBL230	Oxidoreductase		1 / 3
Hydroxycarboxylic acid receptor 2	HCAR2	Q8TDS4	CHEMBL3785	Family A G protein-coupled receptor		20 / 0

SIB Swiss Institute of Bioinformatics

SwissTargetPrediction

Home FAQ Help Download Contact Disclaimer

Query Molecule

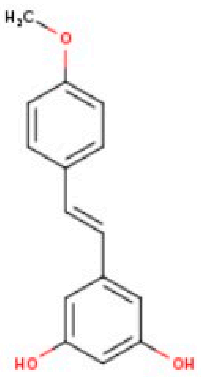

Target Classes

Top 15
Top 25
Top 50
All

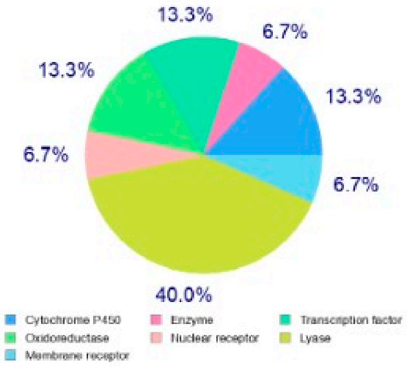

Export results:

Show 15 entries

Search: oxy

Target	Common name	Uniprot ID	ChEMBL ID	Target Class	Probability*	Known actives (3D/2D)
Cyclooxygenase-1	PTGS1	P23219	CHEMBL221	Oxidoreductase	[illegible]	20 / 29
Cyclooxygenase-2	PTGS2	P35354	CHEMBL230	Oxidoreductase	[illegible]	33 / 42
Arachidonate 5-lipoxygenase	ALOX5	P09917	CHEMBL215	Oxidoreductase	[illegible]	83 / 23
11-beta-hydroxysteroid dehydrogenase 1	HSD11B1	P28845	CHEMBL4235	Enzyme	0.0	23 / 3

deoxyrhapontigenin

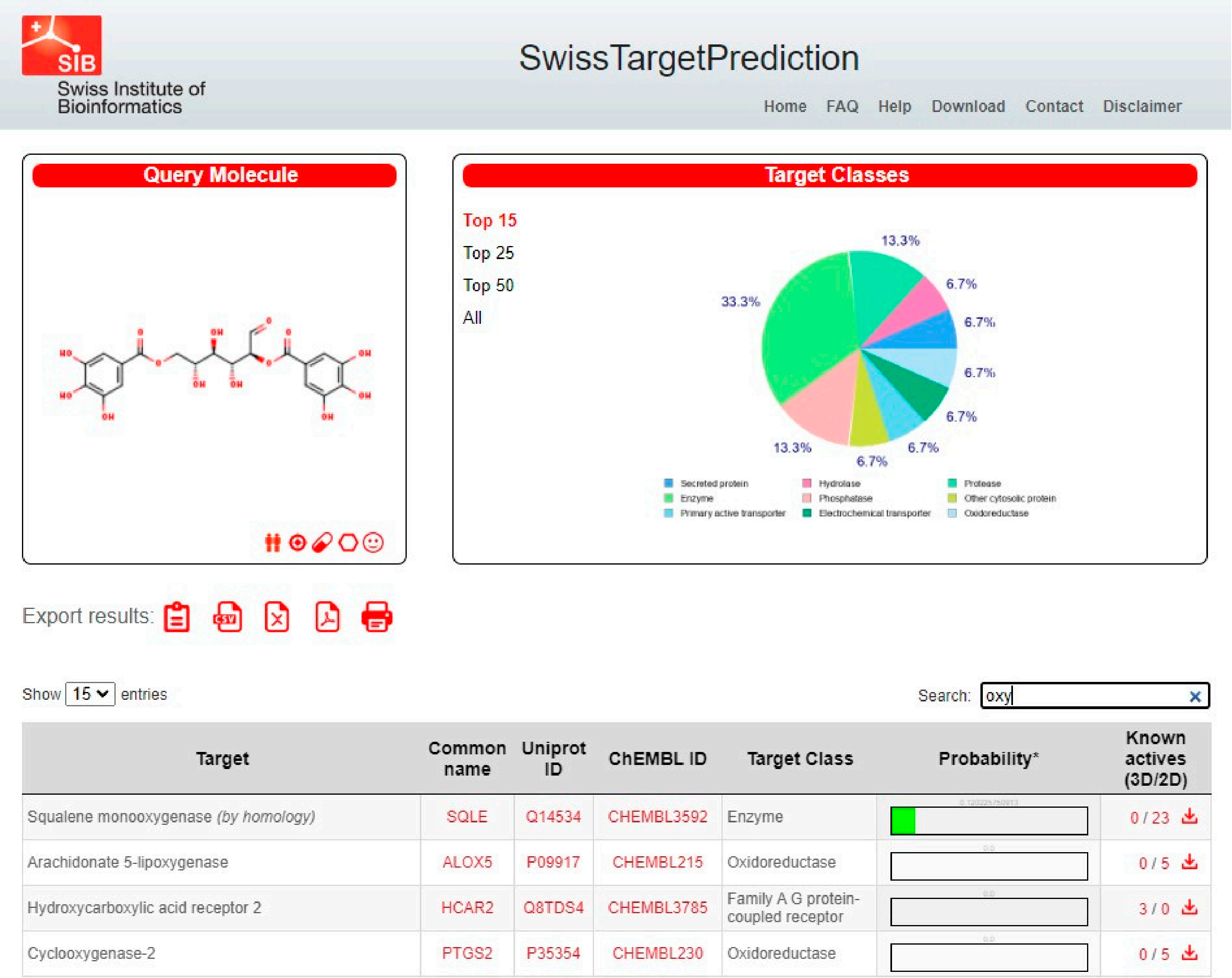

Target	Common name	Uniprot ID	ChEMBL ID	Target Class	Probability*	Known actives (3D/2D)
Squalene monooxygenase *(by homology)*	SQLE	Q14534	CHEMBL3592	Enzyme		0 / 23
Arachidonate 5-lipoxygenase	ALOX5	P09917	CHEMBL215	Oxidoreductase		0 / 5
Hydroxycarboxylic acid receptor 2	HCAR2	Q8TDS4	CHEMBL3785	Family A G protein-coupled receptor		3 / 0
Cyclooxygenase-2	PTGS2	P35354	CHEMBL230	Oxidoreductase		0 / 5

digalloyl glucoside

SIB Swiss Institute of Bioinformatics

SwissTargetPrediction

Home FAQ Help Download Contact Disclaimer

Query Molecule

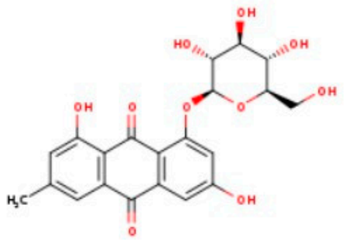

Target Classes

Top 15
Top 25
Top 50
All

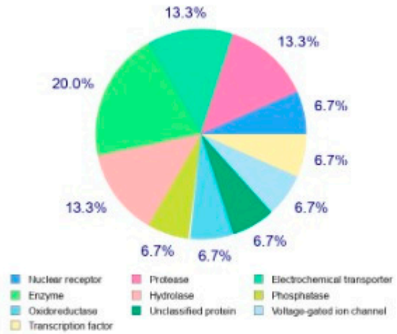

Export results:

Show 15 entries

Search: oxy

Target	Common name	Uniprot ID	ChEMBL ID	Target Class	Probability*	Known actives (3D/2D)
Cyclooxygenase-1 *(by homology)*	Ptgs1	P22437	CHEMBL2649	Enzyme		0 / 2
Cyclooxygenase-2 *(by homology)*	Ptgs2	Q05769	CHEMBL4321	Oxidoreductase		1 / 1

Showing 1 to 2 of 2 entries (filtered from 52 total entries)

Previous 1 Next

*Probability for the query molecule - assumed as bioactive - to have this protein as target.

Swiss Institute of Bioinformatics - © 2022

emodin 8-O-glucoside

SwissDrugDesign | SwissDock SwissParam SwissSidechain SwissBioisostere **SwissTargetPrediction** SwissADME SwissSimilarity | About us

Swiss Institute of Bioinformatics

SwissTargetPrediction

Home FAQ Help Download Contact Disclaimer

Query Molecule

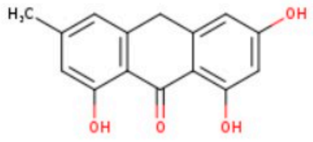

Target Classes

Top 15
Top 25
Top 50
All

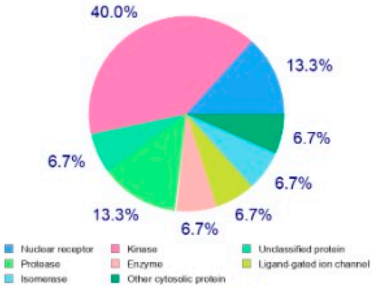

Export results:

Show 15 entries

Search: oxy

Target	Common name	Uniprot ID	ChEMBL ID	Target Class	Probability*	Known actives (3D/2D)
11-beta-hydroxysteroid dehydrogenase 1	Hsd11b1	P50172	CHEMBL3910	Enzyme		12 / 0
Palmitoleoyl-protein carboxylesterase NOTUM *(by homology)*	Notum	Q8R116	CHEMBL3758064	Hydrolase		1 / 0
Arachidonate 5-lipoxygenase-activating protein *(by homology)*	Alox5ap	P30355	CHEMBL3414408	Other cytosolic protein		5 / 0
Hydroxycarboxylic acid receptor 2 *(by homology)*	Hcar2	Q9EP66	CHEMBL4420	Family A G protein-coupled receptor		2 / 0

Showing 1 to 4 of 4 entries (filtered from 100 total entries)

Previous 1 Next

*Probability for the query molecule - assumed as bioactive - to have this protein as target.

emodin anthrone

SIB Swiss Institute of Bioinformatics

SwissTargetPrediction

Home FAQ Help Download Contact Disclaimer

Query Molecule

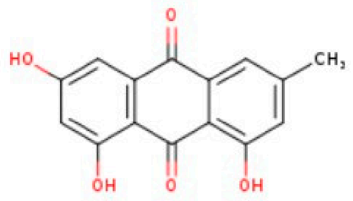

Target Classes

Top 15
Top 25
Top 50
All

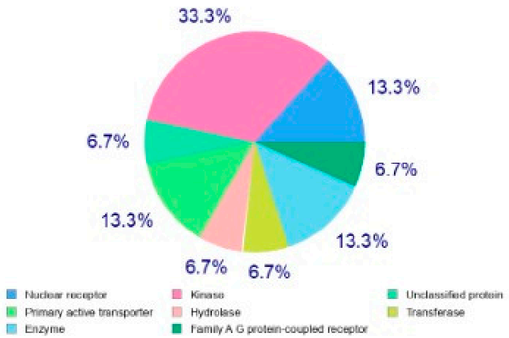

Export results:

Show 15 entries

Search: oxy

Target	Common name	Uniprot ID	ChEMBL ID	Target Class	Probability*	Known actives (3D/2D)
Cyclooxygenase-2 *(by homology)*	Ptgs2	Q05769	CHEMBL4321	Oxidoreductase		2 / 4
Cyclooxygenase-1 *(by homology)*	Ptgs1	P22437	CHEMBL2649	Enzyme		0 / 3
Arachidonate 5-lipoxygenase *(by homology)*	Alox5	P48999	CHEMBL5211	Oxidoreductase		4 / 5
Indoleamine 2,3-dioxygenase 1 *(by homology)*	Ido1	P28776	CHEMBL1075294	Enzyme		0 / 1
Arachidonate 12-lipoxygenase	Alox12	P39655	CHEMBL3225	Enzyme		9 / 3
Carboxylesterase 2 *(by homology)*	Ces2c	Q91WG0	CHEMBL2217	Enzyme		1 / 1

emodin anthrone

SIB Swiss Institute of Bioinformatics

SwissTargetPrediction

Home FAQ Help Download Contact Disclaimer

Query Molecule

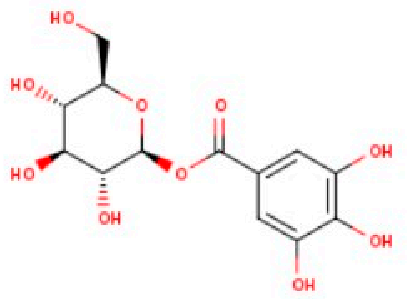

Target Classes

Top 15
Top 25
Top 50
All

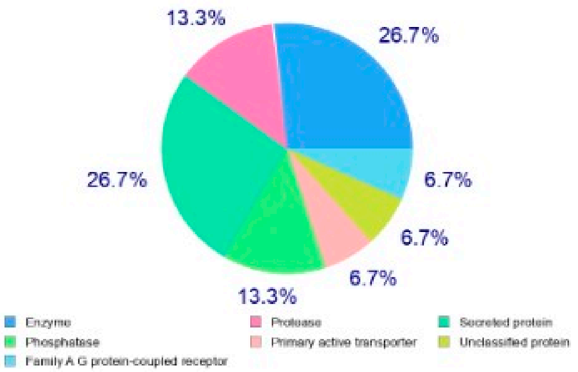

Export results:

Show 15 entries

Search: oxygen

Target	Common name	Uniprot ID	ChEMBL ID	Target Class	Probability*	Known actives (3D/2D)
Squalene monooxygenase *(by homology)*	SQLE	Q14534	CHEMBL3592	Enzyme		0 / 23
Indoleamine 2,3-dioxygenase	IDO1	P14902	CHEMBL4685	Enzyme		1 / 0
Arachidonate 12-lipoxygenase	ALOX12	P18054	CHEMBL3687	Enzyme		1 / 0

glucagallin

SwissTargetPrediction

Swiss Institute of Bioinformatics

Home FAQ Help Download Contact Disclaimer

Query Molecule

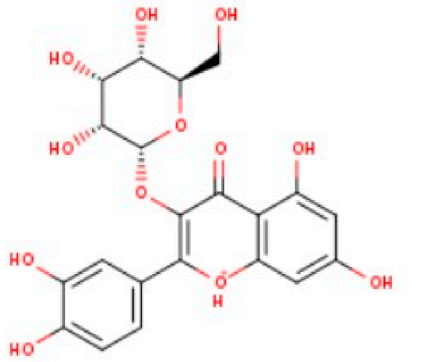

Target Classes

Top 15
Top 25
Top 50
All

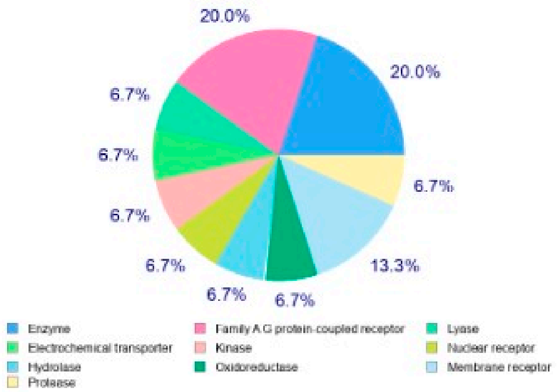

Isoquercitrin

Export results:

Show 15 entries

Search: oxy

Target	Common name	Uniprot ID	ChEMBL ID	Target Class	Probability*	Known actives (3D/2D)
Squalene monooxygenase *(by homology)*	SQLE	Q14534	CHEMBL3592	Enzyme		2 / 0
Cyclooxygenase-1	PTGS1	P23219	CHEMBL221	Oxidoreductase		0 / 2
Cyclooxygenase-2	PTGS2	P35354	CHEMBL230	Oxidoreductase		1 / 1

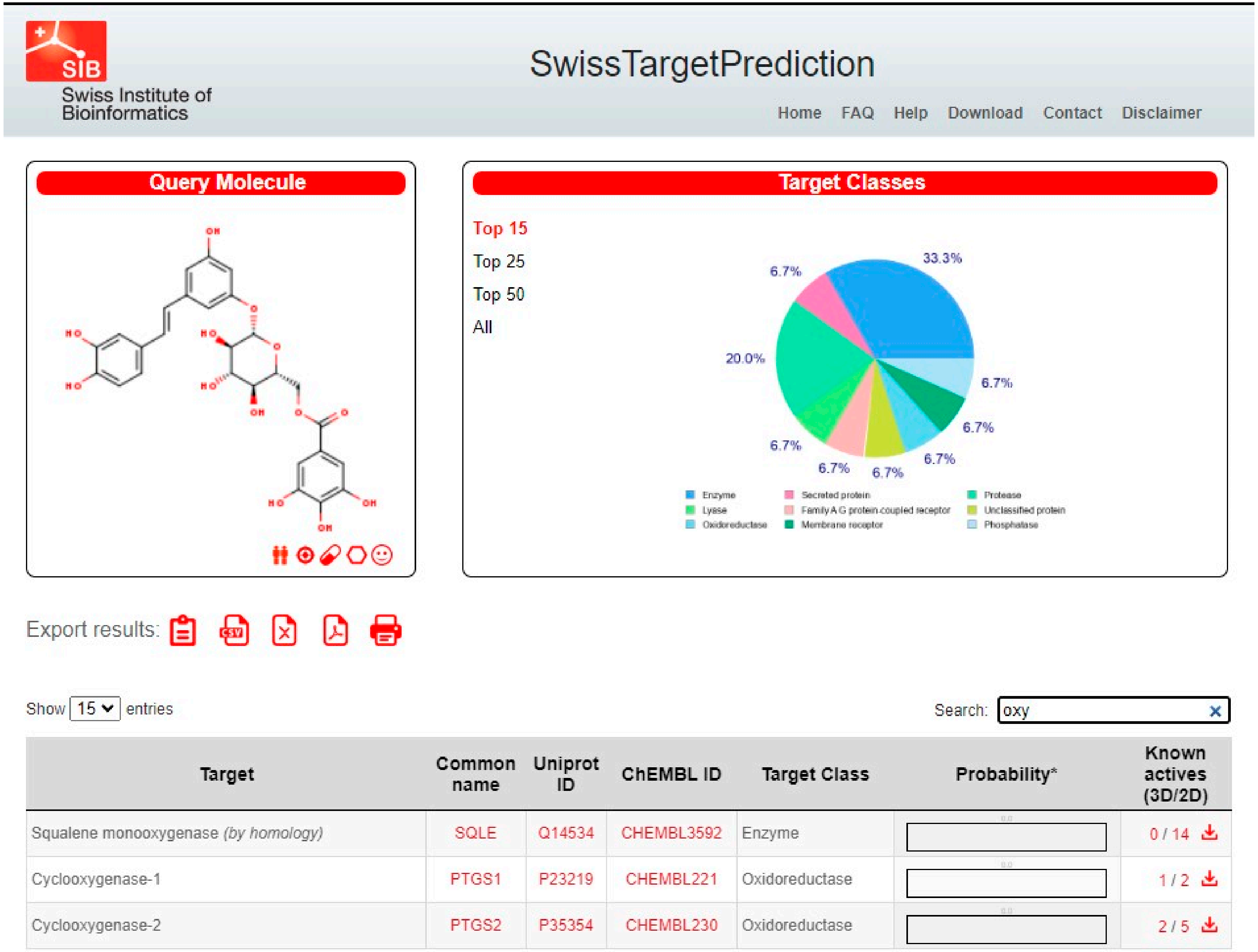

Target	Common name	Uniprot ID	ChEMBL ID	Target Class	Probability*	Known actives (3D/2D)
Squalene monooxygenase *(by homology)*	SQLE	Q14534	CHEMBL3592	Enzyme		0 / 14
Cyclooxygenase-1	PTGS1	P23219	CHEMBL221	Oxidoreductase		1 / 2
Cyclooxygenase-2	PTGS2	P35354	CHEMBL230	Oxidoreductase		2 / 5

rhapontigenin-galloyl-glucopyranoside

SIB Swiss Institute of Bioinformatics

SwissTargetPrediction

Home FAQ Help Download Contact Disclaimer

Query Molecule

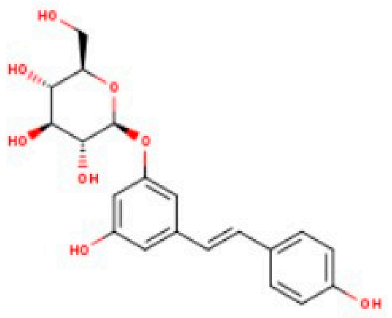

Target Classes

Top 15
Top 25
Top 50
All

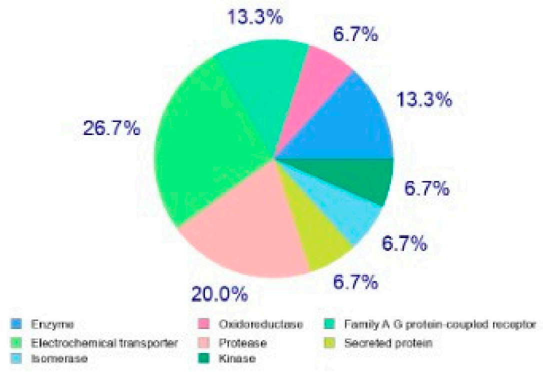

piceid

Export results:

Show 15 entries

Search: oxy

Target	Common name	Uniprot ID	ChEMBL ID	Target Class	Probability*	Known actives (3D/2D)
Hydroxycarboxylic acid receptor 2	HCAR2	Q8TDS4	CHEMBL3785	Family A G protein-coupled receptor		41 / 0
Cyclooxygenase-2	PTGS2	P35354	CHEMBL230	Oxidoreductase		2 / 3
Glutamate carboxypeptidase II	FOLH1	Q04609	CHEMBL1892	Protease		4 / 0
Cyclooxygenase-1	PTGS1	P23219	CHEMBL221	Oxidoreductase		1 / 3

SwissDrugDesign | SwissDock SwissParam SwissSidechain SwissBioisostere **SwissTargetPrediction** SwissADME SwissSimilarity | About us

SIB Swiss Institute of Bioinformatics

SwissTargetPrediction

Home FAQ Help Download Contact Disclaimer

Query Molecule

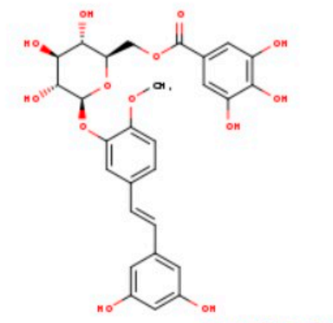

Target Classes

Top 15
Top 25
Top 50
All

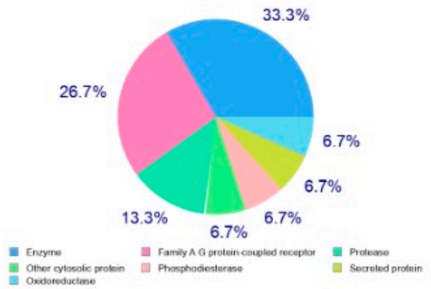

rhapontigenin-galloyl-glucopyranoside

Export results:

Show 15 entries

Search: oxy

Target	Common name	Uniprot ID	ChEMBL ID	Target Class	Probability*	Known actives (3D/2D)
Squalene monooxygenase *(by homology)*	SQLE	Q14534	CHEMBL3592	Enzyme		0 / 14
Hydroxycarboxylic acid receptor 2	HCAR2	Q8TDS4	CHEMBL3785	Family A G protein-coupled receptor		62 / 0
Cyclooxygenase-2	PTGS2	P35354	CHEMBL230	Oxidoreductase		7 / 9

Showing 1 to 3 of 3 entries (filtered from 100 total entries)

Previous 1 Next

*Probability for the query molecule - assumed as bioactive - to have this protein as target.

Swiss Institute of Bioinformatics - © 2022

SwissDrugDesign | SwissDock SwissParam SwissSidechain SwissBioisostere **SwissTargetPrediction** SwissADME SwissSimilarity | About us

Swiss Institute of Bioinformatics

SwissTargetPrediction

Home FAQ Help Download Contact Disclaimer

Query Molecule

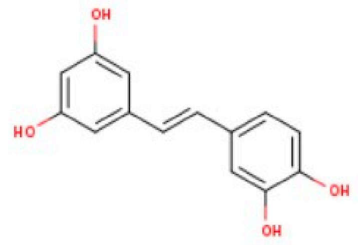

Target Classes

Top 15
Top 25
Top 50
All

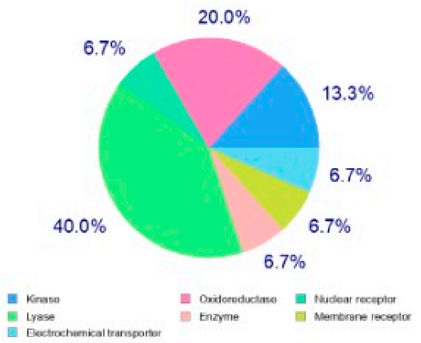

rhapontigenin

Export results:

Show 15 entries

Search: oxy

Target	Common name	Uniprot ID	ChEMBL ID	Target Class	Probability*	Known actives (3D/2D)
Cyclooxygenase-1	PTGS1	P23219	CHEMBL221	Oxidoreductase		9 / 24
Cyclooxygenase-2	PTGS2	P35354	CHEMBL230	Oxidoreductase		10 / 21
Arachidonate 5-lipoxygenase	ALOX5	P09917	CHEMBL215	Oxidoreductase		3 / 19
Arachidonate 15-lipoxygenase	ALOX15	P16050	CHEMBL2903	Enzyme		14 / 0
17-beta-hydroxysteroid dehydrogenase 14	HSD17B14	Q9BPX1	CHEMBL3712868	Enzyme		1 / 0

SIB Swiss Institute of Bioinformatics

SwissTargetPrediction

Home FAQ Help Download Contact Disclaimer

Query Molecule

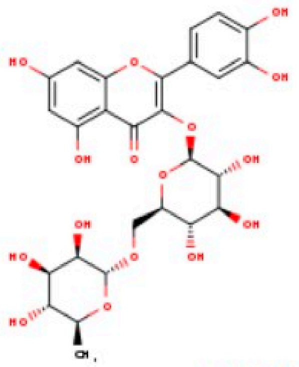

Target Classes

Top 15
Top 25
Top 50
All

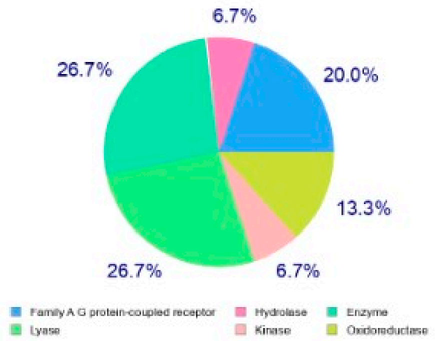

rutin

Export results:

Show 15 entries

Search: oxy

Target	Common name	Uniprot ID	ChEMBL ID	Target Class	Probability*	Known actives (3D/2D)
Cyclooxygenase-2	PTGS2	P35354	CHEMBL230	Oxidoreductase		1 / 4
Arachidonate 5-lipoxygenase	ALOX5	P09917	CHEMBL215	Oxidoreductase		0 / 45
Squalene monooxygenase *(by homology)*	SQLE	Q14534	CHEMBL3592	Enzyme		2 / 0
Arachidonate 15-lipoxygenase	ALOX15	P16050	CHEMBL2903	Enzyme		0 / 3
Arachidonate 12-lipoxygenase	ALOX12	P18054	CHEMBL3687	Enzyme		0 / 2

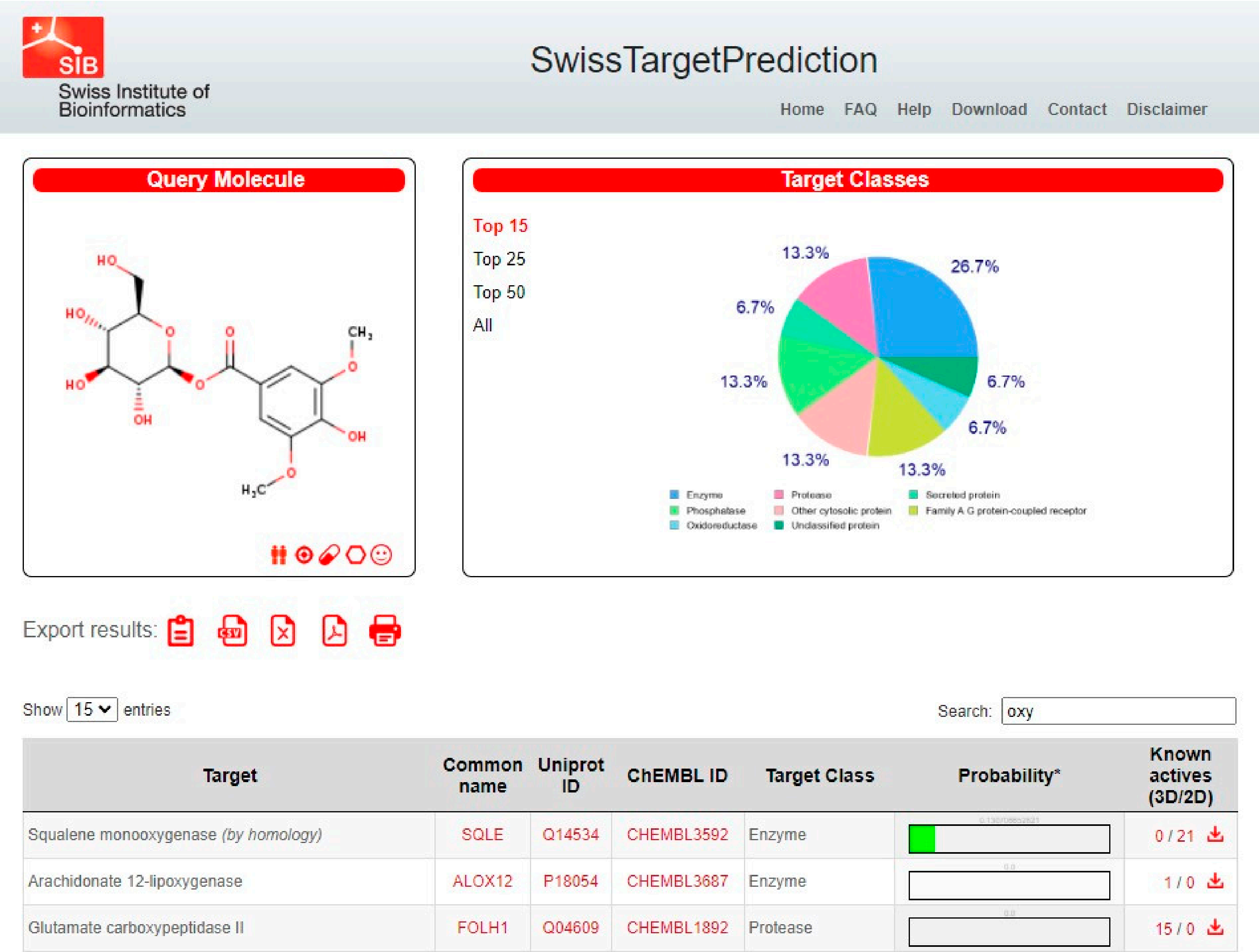

Target	Common name	Uniprot ID	ChEMBL ID	Target Class	Probability*	Known actives (3D/2D)
Squalene monooxygenase *(by homology)*	SQLE	Q14534	CHEMBL3592	Enzyme		0 / 21
Arachidonate 12-lipoxygenase	ALOX12	P18054	CHEMBL3687	Enzyme		1 / 0
Glutamate carboxypeptidase II	FOLH1	Q04609	CHEMBL1892	Protease		15 / 0

syringoyl 1-*O*-glucopyranoside

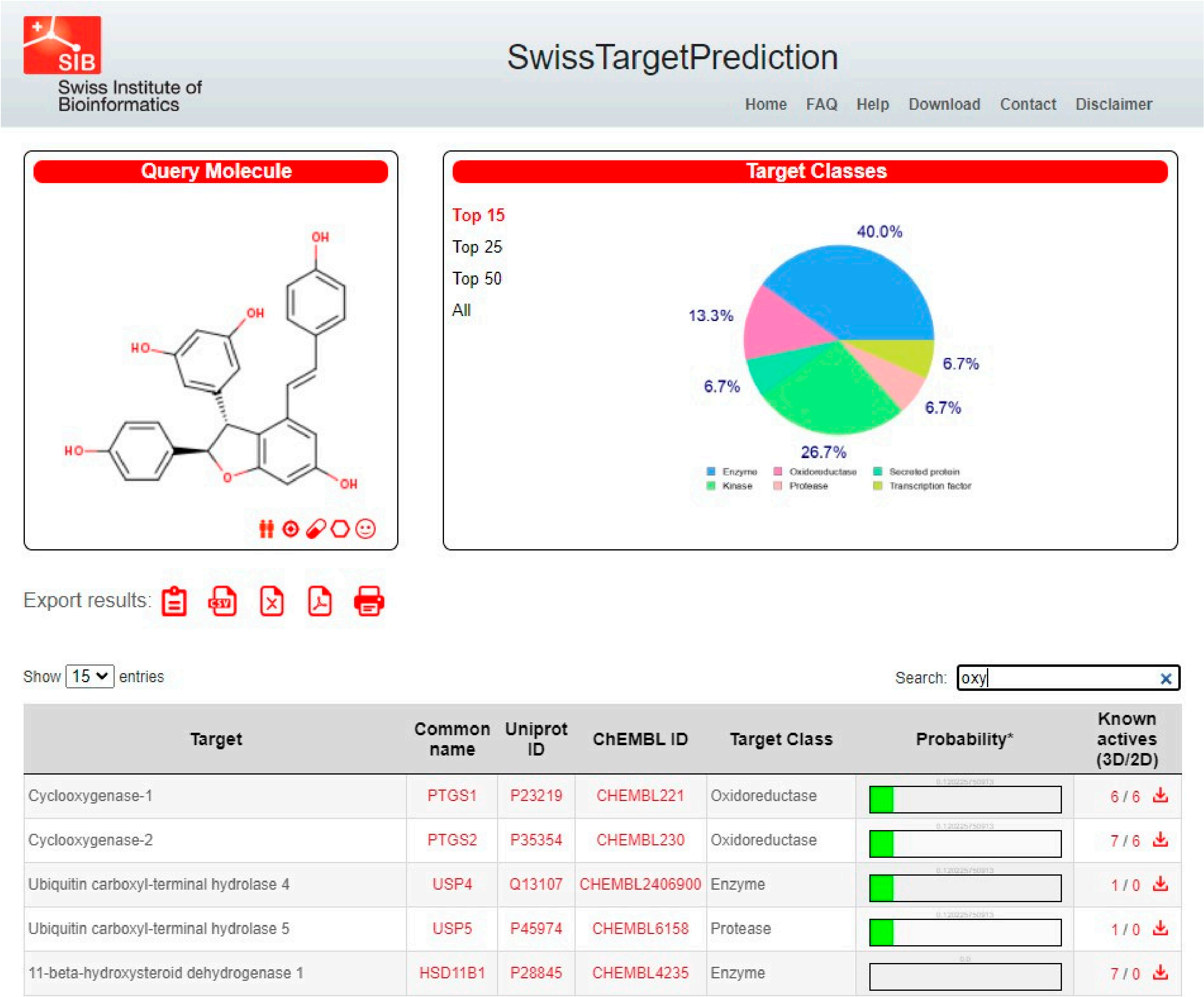

Target	Common name	Uniprot ID	ChEMBL ID	Target Class	Probability*	Known actives (3D/2D)
Cyclooxygenase-1	PTGS1	P23219	CHEMBL221	Oxidoreductase		6 / 6
Cyclooxygenase-2	PTGS2	P35354	CHEMBL230	Oxidoreductase		7 / 6
Ubiquitin carboxyl-terminal hydrolase 4	USP4	Q13107	CHEMBL2406900	Enzyme		1 / 0
Ubiquitin carboxyl-terminal hydrolase 5	USP5	P45974	CHEMBL6158	Protease		1 / 0
11-beta-hydroxysteroid dehydrogenase 1	HSD11B1	P28845	CHEMBL4235	Enzyme		7 / 0

viniferin (resveratrol-dehydrodimer)

Figure S5. The estimation of the most probable macromolecular targets of rhubarb compounds. According to the tool description “The prediction is founded on a combination of 2D and 3D similarity with a library of 370000 known actives on more than 3000 proteins from three different species”. The webtool (http://www.swisstargetprediction.ch/ accessed on 15 October 2022) is described in the article: SwissTargetPrediction: updated data and new features for efficient prediction of protein targets of small molecules, Daina A., Michielin O., Zoete V. Nucleic Acids Res. 2019;47(W1):W357-W364. doi: 10.1093/nar/gkz382. (2019).
